# Safety and efficacy of umbilical cord-derived stem cell therapy for the treatment of cerebral palsy patients: a systematic review

**DOI:** 10.1186/s13023-025-04029-z

**Published:** 2025-10-17

**Authors:** Nader Salari, Fatemeh Morddarvanjoghi, Amin Hosseinian-Far, Faranak Aghaz, Kamran Mansouri, Razie Hasheminezhad, Masoud Mohammadi

**Affiliations:** 1 Department of Biostatistics, School of Health, Kermanshah University of Medical Sciences, Kermanshah, Iran; 2 Sleep Disorders Research Center, Kermanshah University of Medical Sciences, Kermanshah, Iran; 3 Student Research Committee, Kermanshah University of Medical Sciences, Kermanshah, Iran; 4 Department of Business Analytics & Systems, University of Hertfordshire, Hatfield, AL10 9EU UK; 5 Fertility and Infertility Research Center, Health Technology Institute, Kermanshah University of Medical Sciences, Kermanshah, Iran; 6 Nano Drug Delivery Research Center, Faculty of Pharmacy, Health Technology Institute, Kermanshah University of Medical Sciences, Kermanshah, Iran; 7 Medical Biology Research Centre, Kermanshah University of Medical Sciences, Kermanshah, Iran; 8 Research Center for Social Determinants of Health, Jahrom University of Medical Sciences, Jahrom, Iran

**Keywords:** Safety, Effectiveness, Stem cells, Cerebral palsy, Umbilical

## Abstract

**Background:**

Allogeneic umbilical cord blood is regarded as a beneficial source of stem cells with varying therapeutic potential. On the other hand, cerebral palsy is one of the neurological conditions that are the primary contributor to early childhood disability. The aim of this systematic review was to harvest data from currently available sources to determine the safety and efficacy of treating cerebral palsy patients with stem cells obtained from allogeneic umbilical cords.

**Methods:**

For this study, systematic searches of the databases PubMed, Scopus, Web of Science, Embase, ScienceDirect, and Google Scholar were conducted with no time Limit until November 2022. Duplicates were found and eliminated after entering the data from the chosen studies into the Endnote reference management program. The remaining studies were assessed in line with the Preferred Reporting Items for Systematic Reviews and Meta-Analyses phases and the inclusion and exclusion criteria (PRISMA). The search was performed using the keywords of Safety, Effectiveness, Stem Cells, Cerebral Palsy, and Umbilical and the (AND) and (OR) operators and their combinations were used to construct the search strategies.

**Results:**

After several assessments and based on the inclusion and exclusion criteria, 31 of the remaining 58 studies were eliminated. The systematic review method included 7 final studies in the end. Based on the reviewed studies, it was reported that umbilical cord blood is currently one of the best sources of adult stem cells that contain cells with a wide range of therapeutic potential. These studies report that allogeneic umbilical cord blood has the potential to treat cerebral palsy and that concomitant administration of recombinant human erythropoietin (EPO), which has neurotrophic properties, may enhance the efficacy of umbilical cord blood. These studies state that pneumonia and irritability have been reported as complications of umbilical cord blood transfusion. These studies reported that administration of stem cells significantly improves motor function. The safety and efficacy of treating cerebral palsy patients with stem cells taken from an allogeneic umbilical cord were reported in all included investigations.

**Conclusion:**

Cerebral palsy has negative consequences on patients’ quality of life, many aspects of the treatment based on allogeneic umbilical cord stem cells remain unknown. Therefore, the optimal dose, the most suitable type of cell, cell identification, and the best administration route should be determined appropriately. The quality of life of patients with cerebral palsy may be negatively impacted, and many details of the allogeneic umbilical cord stem cell therapy are yet unknown. Consequently, it is important to discover the most suitable type of cell, the optimal dose, and the best delivery method.

## Background

Cerebral palsy (CP) is the most common cause of early childhood impairment. Cerebral palsy is a group of conditions that affect movement and posture. It’s caused by damage that occurs to the developing brain, most often before birth. Symptoms appear during infancy or preschool years and vary from very mild to serious [1–3].

About 3 out of every 1000 individuals throughout the world are affected with CP [4]. The annual cost of caring for a patient with cerebral palsy, who is unable to work and pay taxes, is estimated to be around 87 billion dollars for the economies of Australia and the United States [5]. 70% of CP cases have unknown causes, and 20% of those causes in children may be related to preterm, prenatal trauma, or brain hypoxia. Infections and birth abnormalities that happen during fetal development are some additional reasons of this illness [6].

Stem cells are defined as multipotent cells with the ability to self-renew and the ability to differentiate into other cells [7]. Among the sources of stem cells, allogeneic umbilical cord blood (UCB) is currently a valuable source of adult stem cells that contain cells with variable therapeutic potential [8]. UCB, which is produced from monocytes and contains a combination of regulatory T-cells, stem cells, and hematopoietic cells, can influence the environment of neuroinflammation and be used to treat neurological illnesses [9]. In other words, UCB is the most realistic alternative for treating CP due to its established neuroprotective qualities, which include anti-inflammatory and anti-apoptotic effects [10]. Providing the immune system and paracrine signaling to increase cell survival in damaged tissues, encouraging the proliferation of progenitor cells and increasing angiogenesis is achieved through UCB infusion for the treatment of neurological diseases [11]. Moreover, it has been demonstrated that UCB prevents neuroinflammation by reducing microglial activation and T-cell responses [12]. Although the capacity of mesenchymal stem cells (MSC) to regenerate and differentiate into new cells is another proposed mechanism [13], recent studies have reported the limited efficiency of the MSC and have shown that cell migration to the site of injury is not necessary for treatment [14].

To date, many aspects of UCB-based therapy remain unexplored. To ensure the implementation of safe and effective protocols without raising ethical concerns the optimal dose, the optimum cell type, and the best route of administration of UCB need to be identified [11–13]. Considering the therapeutic importance of stem cells, especially allogeneic umbilical cord blood, it was decided to conduct this systematic review with a focus on the safety and efficacy of treatment with stem cells derived from the umbilical cord, for the treatment of cerebral palsy patients. The purpose of this study is to systematically investigate the safety and effectiveness of treatment with stem cells derived from allogeneic umbilical cord for the treatment of cerebral palsy patients, which can be considered as important evidence for the treatment of cerebral palsy globally.

## Materials and methods

In this study, allogeneic cord-derived stem cell therapy for the treatment of cerebral palsy patients was evaluated for its safety and efficacy using data from original investigations. The study was conducted under the PRISMA protocol and reporting guidelines. The search was performed using the keywords of Safety, Effectiveness, Stem Cells, Cerebral Palsy, and Umbilical with no lower time Limit until November 2022. The (AND) and (OR) operators and their combinations were used to construct the search strategies. The searches were made within the databases of PubMed, Web of Science, Google Scholar, Scopus, Embase, and Science Direct. Moreover, each article’s reference list and citations were manually reviewed to confirm that the searches were thorough. To locate pertinent studies, references to earlier research were also examined. Information from the identified studies was transferred into the EndNote reference management software (Clarivate, Philadelphia, PA 19130). Articles that reported the safety and effectiveness of treatment with stem cells derived from the allogeneic umbilical cord for the treatment of cerebral palsy patients were accepted by the inclusion criteria.

In this study, PICO was examined and expressed as follows: The study population (P) includes cerebral palsy patients; the Intervention includes (I) the treatment of cerebral palsy patients; the Comparison includes (C) the comparison of treatment with umbilical cord stem cells and no treatment group; and the Outcome includes the safety and efficacy of umbilical cord-derived stem cell therapy.

### Inclusion and exclusion criteria

Studies were selected based on the following inclusion criteria:

1. Randomized controlled trial and observational studies that reported the safety and effectiveness of treatment with stem cells derived from allogeneic umbilical cord for the treatment of cerebral palsy patients,
2. Articles of which the full text was available,
3. Articles that provided sufficient data, and.
4. Articles that were written in English.

Exclusion criteria were:

1. Case series,
2. Case report,
3. Review studies of any sort,
4. Articles of which the text was not accessible,
5. Duplicate studies, and.
6. Studies that did not provide enough information.

### Study selection

Study selection was performed according to the PRISMA guidelines, and the stages outlined within [14]. Initially, the studies that were repeated in different databases were excluded from this study, and only one copy was retained. The publications were then initially reviewed based on their titles and abstracts, and irrelevant ones were eliminated using inclusion and exclusion criteria. Further irrelevant research was eliminated at this point as the complete texts of the remaining publications were assessed based on the inclusion and exclusion criteria. To avoid bias, all the steps of reviewing sources and extracting data were completed independently by two reviewers. In cases where there was a difference of opinion between two reviewers, the article was examined by a third reviewer.

### Quality assessment

Qualitative evaluation of studies was performed using the CONSORT checklist, which is a suitable tool for qualitative evaluation of interventional studies [15]. This checklist has 25 general items, each with Minor items and a total of 37 minor items. Different sections include: Title and Abstract, Introduction and Background, Methods, Participants, Interventions, Objectives, Consequences, Sample Size, Randomization, How to Assign Participants, Blind Allocation, Execution, Blindness of Study, Statistical Methods, Results, Flow Participants’ presence, sampling method, initial data of the number of people analyzed, consequences and estimates, auxiliary analysis, adverse reactions, explanations, interpretation, generalizability and general evidence. To rate the articles, if each article referred to the items in the checklist, it was given a score of 1, and if it was not mentioned, a score of zero was given. The Minimum and maximum scores in this checklist are 0 and 37, respectively. Studies with a score greater than or equal to 27 were considered high-quality studies, and studies with a score less than or equal to 17 were considered low-quality studies.

### Data extraction

Data extraction was conducted by two members of the research team using a different pre-prepared checklist. This checklist included: the first author’s name, year of publication, study location, study type, sample size, and study results.

## Results

**Fig. 1:**
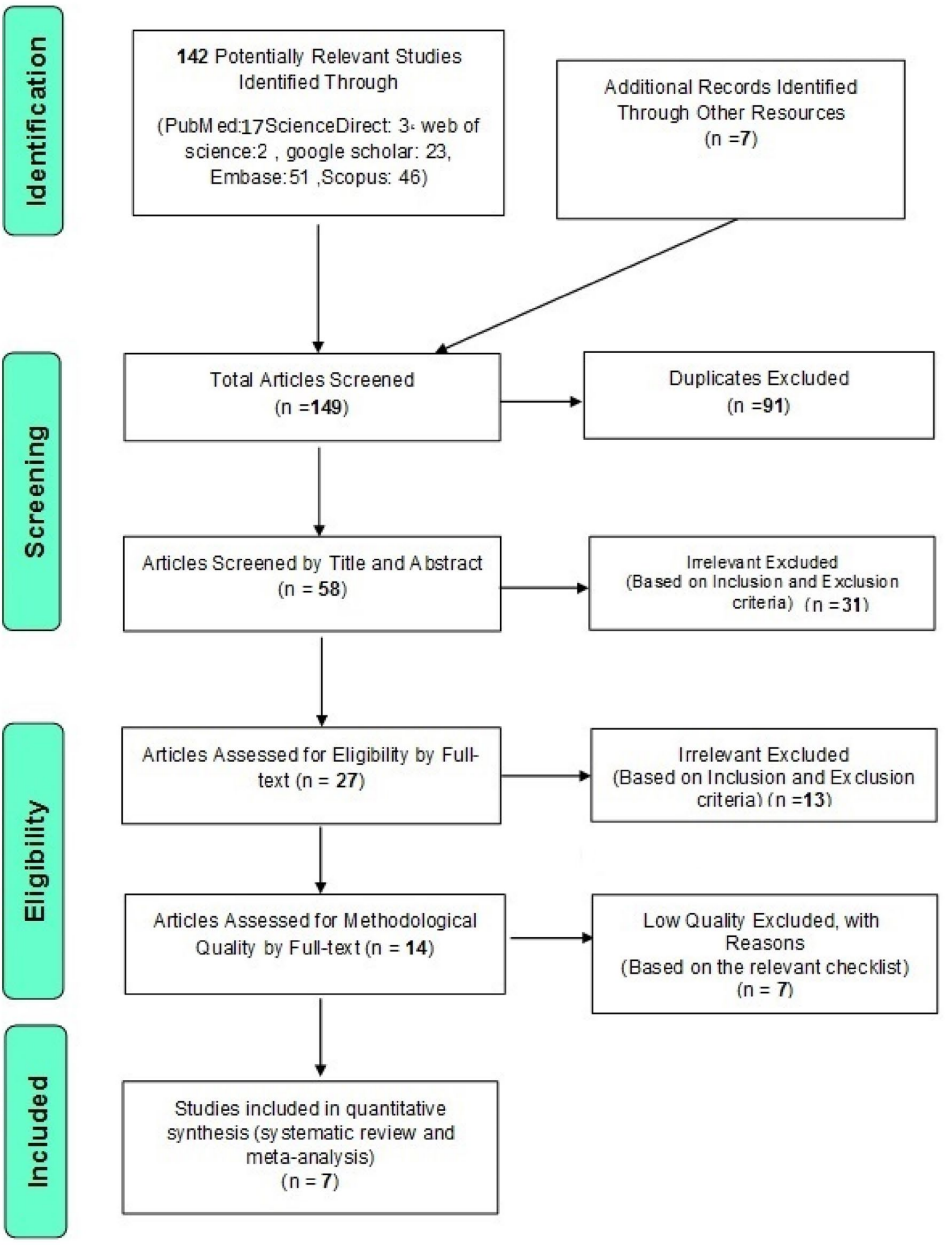
PRISMA Flow Diagram for Study Selection

In this systematic review, reported results of studies that had reported the safety and effectiveness of treatment with stem cells derived from the allogeneic umbilical cord for the treatment of cerebral palsy patients were systematically extracted based on the Guidelines. After searching the aforementioned databases, a total of 142 articles were found; additionally, 7 potentially related articles were chosen by manual search, and their data was also added to the EndNote reference management program. Due to duplication, 91 articles were excluded. In the screening phase, the title and abstract of the studies were evaluated and 31 articles were excluded based on the inclusion and exclusion criteria. In the eligibility evaluation phase, a further 13 articles were excluded through the study of their full text and following the inclusion and exclusion criteria. In the quality evaluation stage, and after examination and review of the full text of the remaining articles and based on the scores obtained from the CONSORT checklist, studies with poor methodological quality were excluded, and finally, 7 studies were included for the final evaluation. Information on these 7 studies is reported in Table 1; Fig. 1. Information on stem cell dose, route of administration, details of immunosuppression, and length of follow-up in the reviewed studies is also reported in Table 2.

**Table 1:** Summary of characteristics, extracted from the included articles that had studied safety and effectiveness of treatment with umbilical cord-derived stem cells for the treatment of cerebral palsy patients

Author	Year	Region	Age	Sample size	Type of study	Result	Instrument
Amanat et al. [16]	2021	Iran	4–14	36	Randomized control	The data showed that cell therapy was clinically effective. + no serious adverse events were reported during the follow-up periods	MAS, PEDI, (CP-QoL)
Feng et al. [20]	2015	Chinese	5.85 ± 6.12	47	A Retrospective Study	had reported adverse events, but no casualties occurred	Statistical analyses were performed using SPSS 17.0 (SPSS Inc., Chicago, IL, USA)
Zarrabi et al. [22]	2022	Iran	4–14	72	a randomized double-blind sham-controlled clinical trial	This trial showed that intrathecal injection of UCB were safe and effective in children with CP.	(GMFM)−66,(MAS), (PEDI), (CP-QoL), (FA), (MD), (CST), (PTR)
Sun et al. [21]	2017	USA	1–6	32	A Randomized, Placebo-Controlled Trial	were well tolerated, and there were no serious adverse events related to the infusions.	Motor function and magnetic resonance imaging brain connectivity studies were performed at baseline, 1-, and 2-years post-treatment.
Moon et al. [18]	2013	South Korea	2–10	32	randomized controlled trial.	Mobilization with G-CSF followed appears to be safe and feasible in CP children.	18.00 software (IBM, 2009)
Kang et al. [19]	20`5	South Korea	6 months to 20 years	36	randomized, placebo-controlled, double-blind trial	no serious adverse events occurred during this study	Muscle strength and gross motor function were evaluated at baseline and 1, 3, and 6 months after treatment. Along with function measurements, each subject underwent 18 F-fluorodeoxyglucose positron emission tomography at baseline and 2 weeks after treatment.
Min et al. [17]	2013	South Korea	36.8 months	31	a double-blind, randomized, placebo-controlled trial.	there were no serious adverse	(GMPM), (BSID-II), (18 F-FDG-PET/CT), (DTI)

**Table 2:** Information on stem cell dose, route of administration, details of immunosuppression, and length of follow-up in the reviewed studies

**Authors**	**Stem cell dose**	**Route of administration**	**Details of the immunosuppression**	**Length of follow-up**
Amanat et al [16]	2 × 10^7^	intrathecal route	No immunosuppressive drugs were used.	from baseline to 12 months after procedures
Feng et al [20]	2-3 × 10^7^	The first injection was intravenous infusion and the rest were intrathecal injections	-	from admission to 6 months after treatment
Zarrabi et al [22]	5 × 10^6^	intrathecal route	No immunosuppressive agents were administered during the course of study	from baseline to 12 months after intervention
Sun et al [21]	1-5 × 10^7^	a single intravenous infusion	-	baseline, 1‐ year, and 2‐years
Moon et al [18]	A single daily dose	subcutaneously	-	5 days
Kang et al [19]	6×10^7^	intra-arterial routes	-	baseline and 1, 3, and 6 months after treatment
Min et al [17]	3 × 10^7^	a single intravenous infusion	-	6 months

Among the selected studies, 2 were conducted in 2013 [17, 18], and 2 in 2015 [19, 20], and the rest of the studies were conducted in the period between 2017 and 2022 [16, 21, 22] (Table 1). Except for 1 study, which was retrospective research [20], the other studies were randomized clinical trials. A total of 286 people with cerebral palsy were examined in these research works. The smallest sample population was observed in the study conducted by Kyunghoon et al., which examined 31 cerebral palsy patients [17]. The largest sample population was also observed in the work of Zarrabi et al., which assessed 72 samples [23]. (Table 1).

Umbilical cord blood (UCB) is now one of the best sources of adult stem cells, containing cells with a range of therapeutic potential [8]. Allogeneic umbilical cord blood has the potential to treat cerebral palsy. Based on the results of the study conducted by Min K et al. in 2013, the simultaneous administration of recombinant human erythropoietin (EPO) which has neurotrophic properties, may boost the efficiency of UCB [17]. Within the same study, UCB therapy improves motor and cognitive impairment in CP children as well as structural and metabolic alterations in the brain [17].

Pneumonia, and irritability were reported as complications of UCB injection. It is also worth mentioning that pneumonia and irritability are also side effects of cyclosporine, as it seems that these complications were brought on by cyclosporine medication rather than by UCB injection (cyclosporine was used in the mentioned studies) [17]. Contrary to the results mentioned in these 2 studies, some articles report the neuroprotective effects of cyclosporine similar to EPO [24–27], which in combination denote stronger neuroprotection [28].

Some other side effects of stem cell injections include fever and vomiting. Intrathecal infusion and commencing treatment in patients 10 years of age or less are risk factors for these side effects [20]. In the first 24 h following treatment, back pain, headaches, and irritability were the most typical side effects, which were largely connected to lumbar puncture [16, 22]. Fever was the most common side effect and this finding was consistent with research that noted the same adverse effect [16–18, 20, 29]. Arrhythmia occurred in one patient during the injection, yet this was resolved spontaneously without any intervention. In addition, six study participants experienced limb numbness which was also resolved spontaneously [16].

Several articles reported that administering stem cells significantly improved motor function [16, 17, 19, 21, 22, 30]. UCB treatment was reported to increase muscle strength in other articles [19, 22]. Moreover, the enhancement of cognitive performance was reported in a clinical trial [17]. Based on the results of 2 other studies, imaging data after UCB injection demonstrated an improvement in the white matter structure (16, 22).

In accordance with these studies, other research reports that UCB injection significantly improves self-care, mobility, and social functioning; the scores on the Cerebral Palsy Quality of Life questionnaire (CP-QOL) in the two domains of “friends and family” and “Participation in activities” were statistically higher than those of the control group; and functional disability and neurological function also improved significantly [23].

It should be noted that, in the study by Min K et al., the treatment group displayed increased activity in the bilateral basal ganglia, thalamus, small areas in the bilateral frontal lobes, the right parietal lobe, and the left temporal lobe, whereas the control group displayed increased activity in a significant area in the bilateral frontal lobes and the basal ganglia [17]. Another study reported that people in the intervention group had a significant decrease in activity in the white matter of the occipital and temporal lobes bilaterally, while an increase in activity was observed in several cortical areas in the frontal and parietal lobes (reference needed). Individuals in the control group showed a decrease in activity in multiple brain regions, yet an increase in activity in small areas of the frontal lobes and parietal cortex was observed [19].

It seems that the prescribed dose of UCB or age variables can affect the therapeutic outcome after injection [17]. When compared to individuals who received lower doses, people who received higher doses demonstrated statistically significant improvement [17, 19, 21]. Moreover, improvements were greater in older CP patients whose motor performance was associated with fewer impairments, while children with more severe physical impairments had greater improvement in mental performance; nevertheless, these improvements were not significant in terms of motor performance [17]. In addition, inherited characteristics can predict the effectiveness of stem cell therapy [31].

All selected studies showed that stem cells derived from the allogeneic umbilical cord do not have serious and life-threatening complications [16–23].

It is worth noting that during a study, a 25-month-old female patient (reference needed) who had deep hypoxia-induced quadriplegia with central gray matter and brainstem involvement died 14 weeks after treatment. The patient was unable to control his or her head due to significant motor impairment. However, tube feeding was required due to poor oral motor function despite that patient’s parents insisted on oral feeding. Moreover, there was persistent obstructive sputum in this patient. From the medical point of view, following the UCB injection, the patient had continuous improvement in neurological function until the last evaluation (within 3 months).

Medical examination revealed that the patient was neurologically stable when presented to the pediatric neurology department for routine seizure follow-up on the day of death. The patient passed away during sleep for no apparent reason, and following a rigorous review of all pertinent documents and incidents, it was found that this patient’s death was not related to UCB injection [17].

## Discussion

Although UCB stem cells have been introduced as a new strategy in the treatment of cerebral palsy, which causes long-term disturbance in patients’ life, it is vital to demonstrate their safety and efficacy. Many studies report that the use of allogeneic umbilical cord stem cells is safe and effective and non-uniform UCB injection can be used for the treatment of CP [32–34]. Possible fundamental mechanisms for the therapeutic effectiveness of UCB stem cells include immune modulation, secretion of nutritional factors, antioxidant metabolites, angiogenic factors, and anti-inflammatory, anti-fibrotic, and anti-apoptotic activity that can increase the repair of injured tissues [35–38]. In trials on CP-affected mice, a preclinical investigation showed that the long-lasting and beneficial benefits of allogeneic UCB injection are caused by their paracrine actions, which accelerate healing in the damaged brain cells and prevent future brain damage [39]. Moreover, clinical trials have demonstrated that both autologous and allogeneic UCBs are beneficial for treating CP patients [17, 40]. The reported effects may be associated with factors other than stem cells. For instance, a significant increase in frontal lobe activity was observed in experiments using EPO. This effect could be induced by dopaminergic stimulation [41, 42], or in another investigation, it was found that vigorous exercise could activate the cerebellum in the untreated control group [43].

Increased metabolism was seen in the thalamus region in the treatment group of a study that utilized UCB; this is a promising finding that may be significant; however, in a prior publication by the same author, UBC was not used, and a decrease in perfusion in the same location (thalamus) was seen [44]. In a different trial that examined the positive benefits of UCB, erythropoietin was also given to the control group in addition to the case group. The disparity in results between the case and control groups may be a result of how UCB affected the brain [17]. It should be highlighted that UCB injection is generally more appropriate in children and adolescents since the cells can be collected safely without invasive and painful procedures. Also, compared to the transplantation of stem cells obtained from autologous bone marrow, the use of allogeneic UCB is cheaper and requires less time. In other words, the isolation, purification, expansion, identification, and extraction of MSCs from bone marrow can take much longer (about 1 month) than the preparation of allogeneic UCB [31].

Another safe and efficient source of stem cells for CP patients is human fetal tissue [45]. However, due to issues with isolation techniques and the requirement for a sophisticated extraction apparatus, this source (embryonic tissue) cannot be employed in many clinical situations [46]. Furthermore, the extraction of cells can lead to fetal death, which raises major ethical concerns. Neural progenitor cells and olfactory lining cells were other types of effective cells used in children with CP and isolated from aborted human embryos [47, 48]. Recent reports suggest that stem cells may be used to treat neurological problems [36]. According to some research, the allogeneic umbilical cord stem cells can treat a variety of neurological conditions [49–52]. Emerging clinical reports have also shown the possibility of using stem cells, including UCB, for pediatric brain lesions [53–55]. According to another study, UCB injection significantly improved gross motor function in individuals with severe cerebral palsy [56]. A non-randomized trial of 8 sets of identical twins (*n* = 16) reported that gross motor function after 1 month of allogeneic intrathecal injection of allogeneic umbilical cord mesenchymal stem cells had no significant improvement during the initial months of treatment, yet after 6 months, significant improvement was observed. In addition, it has been found that the effectiveness rate following UCB injection is predicted by genetics [31]. The overall changes in the average scores of gross motor function were lower in several studies [57, 58, 59]. This could be because of the basic characteristics including the age of the participants. Another study showed that motor development/function among children with CP has more potential after the age of 5–7 [60] and that patients can expect better treatment outcomes at a time that is closer to the moment of injury [61]. In addition, smaller changes in the treatment group could also be due to the single-dose injection of allogeneic umbilical cord mesenchymal stem cells (due to a lower dose or not using consecutive doses) [16].

The most efficient approach to delivering UCB stem cells is unknown, but there are several options available. Some studies reported that intravenous or intra-arterial injection of UCB stem cells can accelerate the functional development of CP patients [17, 19, 62]. Even yet, these methods of cell transplantation are less invasive than intrathecal injection. Studies have shown that UCB cells are less likely to reach lesion sites using intra-arterial/intravenous methods, since they may be retained (trapped) by other organs or the cells are unable to cross the brain’s blood barrier [63, 64].

The intra-arterial injection was also shown to be associated with an increased risk of microembolism (e.g., blood clot) [65]. A case report used a combination of intravenous and intrathecal injections of allogeneic cord mesenchymal cells and reported significant improvements in EEG (electroencephalogram), motor function, and linguistic expressiveness [66]. Although intraventricular cell injection was also suggested, not all patients are candidates for it due to being an invasive treatment [47]. Four studies reported that intravenous/intra-arterial injection of allogeneic umbilical cord mesenchymal cells greatly improved motor function [11, 17, 19, 67]. Another study reported that intravenous injection of allogeneic umbilical cord mesenchymal cells was effective, and those administered with higher matched units (human leukocyte antigen (HLA) with complete HLA or 1 mismatched unit) improved more motor performance than those with 2 mismatched HLA units [67]. In general, the noted complications related to stem cells were not serious or life-threatening, and most of them improved with supportive care or spontaneously.

## Limitation

Future clinical research should compute the normal treatment dose based on the weight or age of the patients in CP to minimize side effects. However, no definitive conclusions can be drawn from small sample trials, and further studies are required to investigate and validate these results. The optimal dose and route of injection should be determined in future studies to find the most appropriate cell transplantation protocol. Only two studies tested different doses (and only 2 different doses) [21, 23]. Longer follow-up periods are recommended to determine the long-term safety and efficacy of treatment with stem cells. The small sample size in some of the selected studies is another limitation of this review.

## Conclusion

According to the research reviewed, these studies reported that UCB stem cells have beneficial effects in the improvement and treatment of cerebral palsy patients, and given that no major side effects were observed in the use of this treatment in the patients studied, it can be considered in the treatment of these patients.

## Data Availability

Datasets are available through the corresponding author upon reasonable request.

## Abbreviations

MAS: Modified Ashworth Scale
PEDI: Pediatric Evaluation of Disability Inventory
CP-QOL: Cerebral Palsy Quality of Life
UCB: Umbilical Cord Blood
GMFM: Gross Motor Function Measure
FA: Fractional Anisotropy
MD: Mean Diffusivity
CST: Corticospinal Tract
PTR: Posterior Thalamic Radiation
G-CSF: Granulocyte Colony Stimulating Factor
GMPM: Gross Motor Performance Measure
BSID-II: Bayley Scales of Infant Development-II
18F-FDG-PET/CT: F-Fluorodeoxyglucose Positron Emission Tomography
DTI: Diffusion Tensor Images
